# STIM1 Reduction Prevents Tubular Aggregate Formation and Compromises Muscle Performance in Ageing Mice

**DOI:** 10.1002/jcsm.70151

**Published:** 2025-12-07

**Authors:** Laura Pérez‐Guàrdia, Roberto Silva‐Rojas, Jocelyn Laporte, Johann Böhm

**Affiliations:** ^1^ Department of Translational Medicine and Neurogenetics, Institute of Genetics and Molecular and Cellular Biology (IGBMC), INSERM U1258, CNRS UMR7104 University of Strasbourg Illkirch France

**Keywords:** ageing, calcium, muscle, STIM1, tubular aggregates

## Abstract

**Background:**

Ageing is an irreversible process involving the gradual decline of cellular functions in all tissues. In male mice, age‐related loss of muscle force is accompanied by the formation of tubular aggregates, which are honeycomb‐like structures composed of membrane tubules, proteins and Ca^2+^ deposits. Tubular aggregates are also found in tubular aggregate myopathy (TAM) and Stormorken syndrome (STRMK), two clinically overlapping human disorders affecting skeletal muscle, bones, skin, spleen and platelets. TAM/STRMK is caused by gain‐of‐function mutations in the ubiquitously expressed Ca^2+^ sensor STIM1 and results in excessive extracellular Ca^2+^ entry and the dysregulation of Ca^2+^ homeostasis.

**Methods:**

To understand the correlation between ageing, tubular aggregate formation, Ca^2+^ and STIM1, we conducted comparative analyses of WT and *Stim1* ^*+/−*^ male mice until 18 months of age. We examined growth, general and specific muscle force, fatigability and muscle structure.

**Results:**

*Stim1* ^*+/−*^ mice were born with the expected Mendelian ratio and showed unremarkable postnatal development with normal body and organ weight. However, at 18 months, *Stim1* ^*+/−*^ mice manifested delayed muscle contraction (*Δ* = 28%, *p* < 0.05) and relaxation (*Δ* = 40%, *p* < 0.01) kinetics as well as exacerbated fatigue (*Δ* = 28%, *p* < 0.05) compared with age‐matched controls. Morphological investigations of *Stim1* ^*+/−*^ muscle sections by light and electron microscopy uncovered a shift towards slow myofibres and mitochondrial proliferation accompanied by enhanced SDH activity (*Δ* = 27%, *p* < 0.0001), an almost twofold increase in ROS production (*p* < 0.05), and signs of mitophagy—all representing histopathological hallmarks of age‐related deterioration of muscle function known as sarcopenia. Strikingly, tubular aggregates—though abundant in WT muscles at 18 months—were absent in *Stim1* ^*+/−*^ mice.

**Conclusions:**

Taken together, STIM1 depletion by 50% had no discernible effect on muscle function in young adult male mice, but compromised muscle performance and resistance to fatigue at later life stages. These findings highlight a critical role of STIM1 and Ca^2+^ balance in the maintenance of muscle physiology, fibre type composition and mitochondrial bioenergetics. The absence of tubular aggregates in *Stim1* ^*+/−*^ mice indicates that tubular aggregates possibly play a protective role and may contribute to the prevention of age‐related muscle alterations.

## 1 Introduction

Tubular aggregate myopathy (OMIM #160565 and #615883) is a hereditary disorder characterized by muscle weakness, cramps and myalgia. Many patients also exhibit one or more multisystemic features including short stature, miosis, thrombocytopenia, ichthyosis, dyslexia and spleen anomalies, and the full clinical picture is referred to as Stormorken syndrome (STRMK; OMIM #185070) [1]. TAM/STRMK is primarily caused by heterozygous missense mutations in *STIM1* and *ORAI1*, encoding key components of store‐operated Ca^2+^ entry (SOCE) [2]. SOCE is a fundamental mechanism controlling Ca^2+^ influx in all cell types and plays a crucial role in the precise regulation of Ca^2+^‐dependent cellular processes including skeletal muscle contraction and relaxation [3].

In skeletal muscle, Ca^2+^ is mainly stored in the sarcoplasmic reticulum (SR) and released into the cytosol via the reticular Ca^2+^ channel RyR1 to initiate muscle contraction. Upon Ca^2+^ store depletion, the Ca^2+^ sensor STIM1 unfolds, oligomerizes and translocates to SR/plasma membrane junctions where the STIM1 oligomers activate the Ca^2+^ channel ORAI1 to trigger extracellular Ca^2+^ entry and enable Ca^2+^ store replenishment [4]. In TAM/STRMK, the *STIM1* and *ORAI1* mutations lead to SOCE overactivation, resulting in excessive extracellular Ca^2+^ influx [5, 6, 7]. Investigations on *Stim1* ^*R304W/+*^ mice harbouring the most common TAM/STRMK mutation have shown that the elevated cellular Ca^2+^ levels impair muscle contraction kinetics and induce reticular Ca^2+^ stress, ultimately leading to myofibre degeneration [8].

In addition to signs of myofibre degeneration, muscle biopsies from TAM/STRMK patients consistently display tubular aggregates as the principal histological anomaly [9]. Tubular aggregates are densely packed membrane tubules containing large amounts of Ca^2+^ and various SR proteins such as RyR1, STIM1 or the Ca^2+^ buffer calsequestrin [10, 11]. It is therefore likely that the aggregates originate from the SR, although the precise mechanisms underlying their formation and their contribution to muscle dysfunction remain elusive.

Notably, tubular aggregates are not restricted to TAM/STRMK, have also been observed in other inherited and acquired muscle disorders, and accumulate in normal muscle with age in inbred (but not outbred) mouse strains [12, 13]. Depending on the genetic background, tubular aggregates can appear as early as 5 months of age in male mice and increase in size and prevalence over time [13]. Unlike TAM/STRMK, the age‐related occurrence of tubular aggregates is not caused by gene mutations affecting the SOCE pathway, but supposedly arises from alterations of SR architecture, Ca^2+^ handling and general cellular homeostasis associated with the ageing process [11, 14]. Nonetheless, tubular aggregates in TAM/STRMK and ageing mice are of comparable structure and appearance [12], suggesting a common pathomechanism.

A hallmark of ageing is sarcopenia, a progressive decline of muscle function characterized by reduced muscle mass, strength and endurance. Multiple factors contribute to sarcopenia, including mitochondrial dysfunction and dyshomeostasis, increase of reactive oxygen species (ROS), impaired autophagy, chronic low‐level inflammation and altered Ca^2+^ signalling pathways [15, 16, 17]. Given the similarities with TAM/STRMK, understanding the role of SOCE in the age‐related deterioration of muscle physiology and morphology is of both scientific and clinical significance.

In this study, we aimed to elucidate the physiopathological link between SOCE, muscle ageing and tubular aggregate formation by modulating STIM1 expression in mice. We examined *Stim1* ^*+/−*^ mice expressing 50% of the normal STIM1 level at 4, 10 and 18 months to assess the impact of reduced SOCE on muscle ageing. Our findings revealed a critical role of STIM1 in the formation of tubular aggregates and the maintenance of muscle morphology and physiology.

## 2 Methods

### 2.1 Animals

Mice were bred and housed in ventilated cages in temperature‐ and humidity‐controlled rooms and pathogen‐free conditions with 12‐h day light/dark cycles and free access to water and food. Floxed *Stim1* ^*L2/+*^ mice (C57BL/6 background) were kindly provided by Anjana Rao (La Jolla Institute for Immunology, University of California San Diego, USA) [18], and the Gt (ROSA)26Sor mice to yield excision of *Stim1* exon 2 (suppl. Figure 1A) were kindly provided by the ICS (Institut Clinique de la Souris, Illkirch, France). Animal care, experimentation and euthanasia were in accordance with French and European guidelines, approved by the Institutional Ethics Committee Com'eth (Project Numbers APAFIS #2016031110589922, #202052516535988 and #2019062813376603), and accredited by the French Ministry for Superior Education and Research (MESR) in accordance with the Directive of the European Parliament (2010/63/EU) on the protection of animals used for scientific purposes. Experiments were performed on male and female (muscle histology only) WT and *Stim1* ^*+/−*^ mice (C57BL/6N) at 4, 10 and 18 months of age. Genotyping primers are listed in Table S1.

### 2.2 DNA Quantification and Gene Expression

Genomic DNA was extracted from frozen muscle tissue using a lysis buffer (100 mM Tris–HCl pH 8.0, 200 mM NaCl, 5 mM EDTA, 0.2% SDS) supplemented with proteinase K. DNA pellets were resuspended in Tris‐EDTA buffer solution (10 mM Tris–HCl pH 7.5, 1 mM EDTA) and quantified using a NanoDrop spectrophotometer (Thermo Fisher Scientific, Waltham, Massachusetts, USA). *Nd1* and *Rsp11* served as reference genes to quantify mitochondrial and nuclear DNA, respectively. Primer sequences are listed in Table S1.

RNA was extracted from frozen muscle samples by mechanical lysis with a Precellys homogenizer (Bertin Technologies, Montigny‐le‐Bretonneux, France) in TRIzol reagent (ThermoFisher Scientific). RNA concentration was determined by spectrophotometry (Nanodrop 2000, ThermoFisher Scientific), and cDNA was generated using SuperScript IV Reverse Transcriptase (ThermoFisher Scientific). For qRT‐PCR, the cDNA was amplified using the SYBR Green Master Mix I and expression levels were quantified on a LightCycler 480 Real‐Time PCR System (both Roche Diagnostics, Basel, Switzerland). *Rsp11* served as a reference gene. Primer sequences are listed in Table S1.

### 2.3 Protein Studies

For protein extraction, tibialis anterior and soleus samples were lysed in radio immunoprecipitation (RIPA) buffer supplemented with 1 mM PMSF, 1 mM DTT and complete mini EDTA‐free protease inhibitor cocktail (Roche), loaded on a 10% SDS‐PAGE gel, and transferred on nitrocellulose membranes using the Transblot TurboTM RTA Transfer Kit (Biorad, Hercules, California, USA). Membranes were blocked in Tris‐buffered saline (TBS) containing 5% nonfat milk and 0.1% Tween 20. Ponceau S staining (Sigma‐Aldrich, St. Louis, Missouri, USA) served as a loading control.

The following primary and secondary antibodies were used for western blot and immunofluorescence on tibialis anterior, gastrocnemius and soleus samples: donkey anti‐goat IgG‐HRP (Jackson Immunoresearch, West Grove, Pennsylvania, USA), goat anti‐mouse IgG‐HRP (115‐036‐068, Jackson Immunoresearch), goat anti‐mouse IgG1 Alexa 488 (115‐545‐205, Jackson ImmunoResearch), goat anti‐mouse IgG2b Cy3 (115‐165‐207, Jackson ImmunoResearch), goat anti‐mouse IgM DyLight 405 (115‐475‐075, Jackson ImmunoResearch), goat anti‐rabbit IgG‐HRP (AB‐20.0385, Jackson Immunoresearch), mouse anti‐DHPR (ab2862, Abcam, Cambridge, UK), mouse anti‐OPA1 (612606, Sigma‐Aldrich), mouse anti‐MHC I (BA‐D5, DSHB, University of Iowa, USA), mouse anti‐MHC IIa (SC‐71, DSHB), mouse anti‐MHC IIb (BF‐F3, DSHB), mouse anti‐SERCA1 (MA3‐911, ThermoFisher Scientific), mouse anti‐triadin (Sigma‐Aldrich), rabbit anti‐Caveolin‐3 (ab171752, Abcam), rabbit anti‐FIS1 (GTX111010, Sigma‐Aldrich), rabbit anti‐SOD1 (NBP2‐24915, Novus Biologicals, Centennial, Colorado, USA), rabbit anti‐SOD2 (13194, Cell Signalling Technology, Danvers, Massachusetts, USA), rabbit anti‐STIM1 (AB9870, Millipore, Burlington, Massachusetts, USA), rabbit anti‐TOM20 (AB‐20.0374, Abcam) and wheat germ agglutinin Alexa 647 (W32466, ThermoFisher Scientific). DAPI (ThermoFisher Scientific) was used to stain nuclei. To determine ROS production, muscle sections were treated with dihydroethidium (DHE, ThermoFisher Scientific), and the average signal intensity of DHE converted into ethidium was quantified on a region of interest (ROI) using the ImageJ software. Images were acquired using an Axioserver microscope (Zeiss, Jena, Germany).

### 2.4 Muscle Morphology

For histological analyses, tibialis anterior, gastrocnemius and soleus samples were frozen in nitrogen‐cooled isopentane and 8‐μm transversal sections were stained with haematoxylin/eosin (HE), Gomori trichrome, succinic dehydrogenase (SDH) and ATPase to assess general muscle structure, the occurrence of tubular aggregates, mitochondrial activity and fibre type distribution. Images were recorded with the Nanozoomer 2HT slide scanner (Hamamatsu, Japan).

For electron microscopy, muscle specimens were fixed in a solution containing 2.5% paraformaldehyde and 50 mM Ca^2+^ in cacodylate buffer (0.1 M, pH 7.4), immersed in cacodylate buffer, postfixed in 1% osmium tetroxide in 0.1 M cacodylate, dehydrated in graded alcohol (50%, 70%, 90% and 100%) and propylene oxide, and embedded in Epon 812 resin. Ultrathin 70‐nm sections were examined on a Morgagni 268D electron microscope (FEI, Electron Optics, Eindhoven, the Netherlands), and digital images were captured with a Mega View III Camera (Soft Imageing System, Münster, Germany).

### 2.5 General Muscle Force

To assess general muscle force, mice were suspended upside down to a cage grid for a maximum of 60 s, and the time to fall (=hanging time) was recorded. The four‐paw grip strength was assessed with a dynamometer (Bioseb, Vitrolles, France). Both hanging and grip tests were performed in triplicate with a 5‐ to 10‐min rest interval.

### 2.6 In Situ Muscle Force

To assess in situ muscle force, 4‐, 10‐ and 18‐month‐old mice were anaesthetized by intraperitoneal injections of domitor/fentanyl (2/0.28 mg/kg) mix, diazepam (8 mg/kg) and fentanyl (0.28 mg/kg). The tibialis anterior was partially excised and the distal tendon was attached to an isometric force transducer (Complete1300A Mouse Test System, Aurora Scientific, Aurora, Canada). Maximal force was determined through incremental electrical pulses of the sciatic nerve ranging from 1 to 125 Hz spaced by 30 s and divided by the cross‐sectional area (corresponding to muscle mass [mg]/optimal length [mm] × mammalian muscle density [1.06 mg/mm^2^]) to obtain specific muscle force.

Muscle contraction (time to reach 100% muscle force) and relaxation (time to decrease muscle force by 50%) were measured after single stimulations from the 1 Hz stimulation starting the force‐frequency train. Fatigue was assessed through continuous stimulations of 20 s and 50 Hz or 80 stimulations of 2 s and 50 Hz, and time to fatigue is defined as the time until maximal force declines by 50%.

### 2.7 Ca^2+^ Measurements

Primary myoblasts were isolated from 5‐day‐old mice, plated on Matrigel Reduced Factor‐coated plates (Corning Life Sciences, Corning, New York, USA), and cultured in Iscove's Modified Dulbecco's Medium (IMDM, ThermoFisher Scientific) supplemented with 20% FCS (fetal calf serum) and 1% CEE (chicken embryo extract). Cells were differentiated into myotubes at 70% confluency using IMDM with 2% horse serum.

Four days after differentiation, cells were incubated in Ringer solution containing 2 mM Ca^2+^ and 5 μM Fura‐8TM (AAT Bioquest, Pleasanton, California, USA). Resting cytosolic Ca^2+^ was assessed in 2 mM Ca^2+^ Ringer solution, and SOCE activity in Krebs‐Ringer solution containing 10 mM Ca^2+^. Fura‐8 fluorescence ratio was recorded on a SP8 UV confocal microscope (Leica, Wetzlar, Germany), and data analyses were performed with ImageJ. SOCE amplitude reflects the difference in maximal Fura‐8 ratio before and after the switch from Ca^2+^‐free to Ca^2+^‐containing Krebs‐Ringer solution.

### 2.8 Statistical Analysis

All animal experiments were conducted in a blinded manner. Statistical analyses were performed using the GraphPad Prism program 10.0.2 (GraphPad Software, San Diego, California, USA). Data are expressed as mean ± standard error of the mean (SEM) and data distribution was verified using the Shapiro–Wilk test. For the comparison of two groups of normal or lognormal data, the unpaired Student's *t* test with or without Welch's correction was applied. For nonnormal or lognormal data, the Mann–Whitney statistical test was used. For the comparison of more than two groups, normally distributed data were analyzed with one‐way ANOVA followed by Tukey's multiple comparison test, and for nonnormally distributed data, the Kruskal–Wallis test followed by Dunn's multiple comparison test was used. Significant differences are indicated as *p < 0.05, **p < 0.01, ***p < 0.001 and ****p < 0.0001.

## 3 Results

### 3.1 STIM1 Reduction Has No Major Pathogenic Effect on Overall Health and Life Span but Prevents Tubular Aggregate Formation

To decipher the implication of STIM1 and SOCE in ageing and the age‐related occurrence of tubular aggregates in rodents, we examined the overall physiology of *Stim1* ^*+/−*^ mice [18] and investigated muscle function and structure until late adulthood.

**FIGURE 1:**
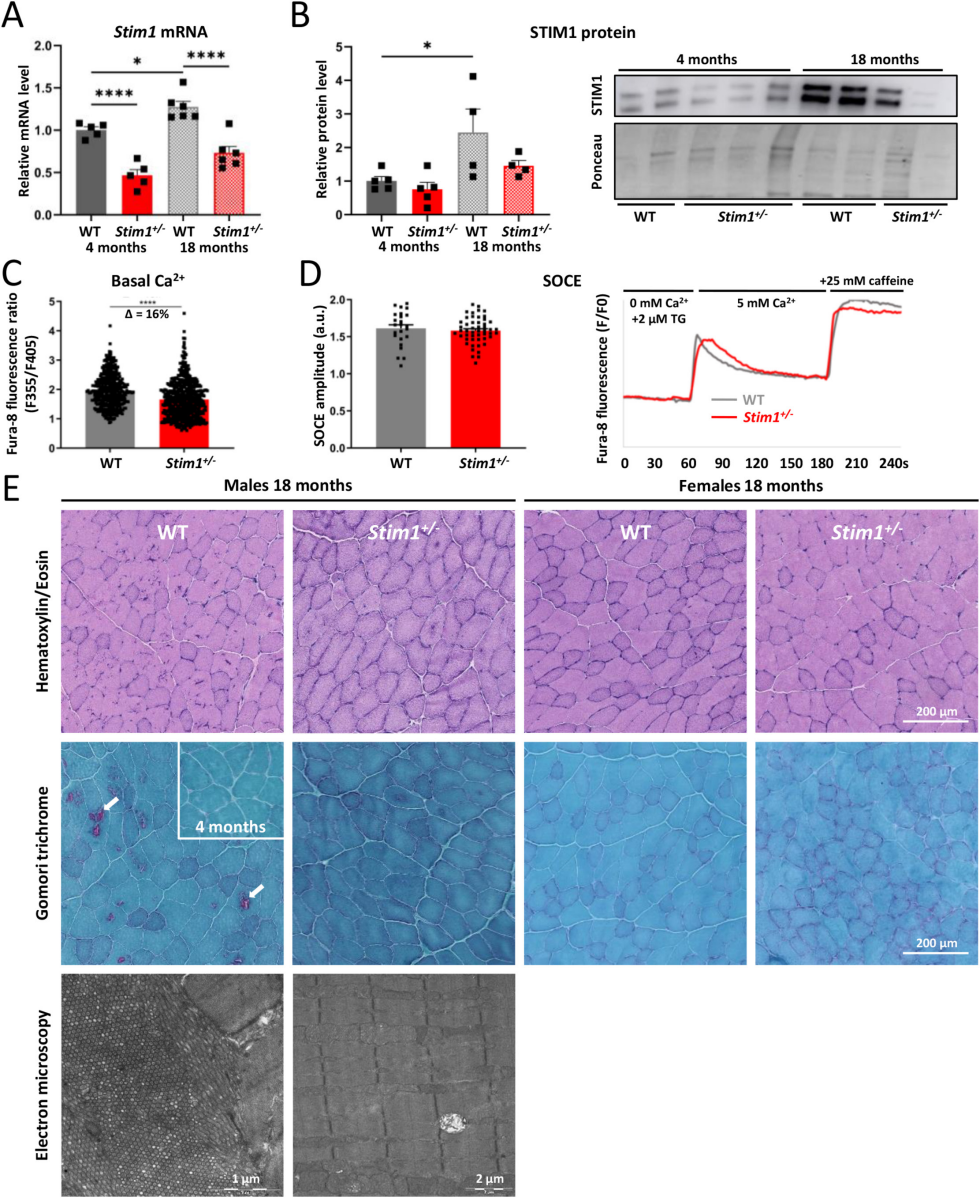
Normal overall development and absence of tubular aggregates in *Stim1* ^*+/−*^ mice. (A) Compared with WT littermates, *Stim1* expression was reduced by half in *Stim1* ^*+/−*^ mice at 4 and 18 months. Note that *Stim1* expression increased with age in WT and *Stim1* ^*+/−*^ mice. (B) Western blot and quantification of band intensities evidenced reduced STIM1 protein levels in *Stim1* ^*+/−*^ mice at 4 and 18 months compared with WT controls. STIM1 levels increased with age in both genotypes. (C) Basal cytosolic Ca^2+^ levels were reduced in *Stim1* ^*+/−*^ myotubes compared with WT controls. (D) Quantification of SOCE activity and representative traces of cytosolic Ca^2+^ levels after subsequent addition of 2 μM thapsigargin (TG), 5 mM Ca^2+^ and 25 mM caffeine showed comparable levels of extracellular Ca^2+^ entry in WT and *Stim1* ^*+/−*^ myotubes. (E) Histological and ultrastructural examination of tibialis anterior sections from 18‐month‐old mice. Tubular aggregates appeared in red on Gomori trichrome stain (arrows) and as densely packed membrane tubules by electron microscopy in 18‐month‐old WT males and were absent in WT females and *Stim1* ^*+/−*^ mice. The inset illustrates the absence of tubular aggregates in WT males at 4 months. Data are presented as mean values ± SEM. For the comparison of two groups, the unpaired Student's *t* test with or without Welch's correction was applied, and for the comparison of more than two groups, data were analyzed with one‐way ANOVA followed by Tukey's multiple comparison test. Significant differences are indicated as *p < 0.05 and ****p < 0.0001.

*Stim1* ^*+/−*^ mice were born with the expected Mendelian ratio (suppl. Figure 1B), were fertile, and showed normal body mass, body length and survival rate until 18 months of age (suppl. Figure 1C,D). In agreement, the organ mass of the spleen, liver and diverse slow‐ and fast‐twitch muscles including soleus, tibialis anterior, diaphragm and gastrocnemius was comparable in *Stim1* ^*+/−*^ mice and WT littermates (suppl. Figure 1E). RT‐qPCR on muscle samples from *Stim1* ^*+/−*^ mice revealed reduced *Stim1* expression at both 4 and 18 months compared with WT controls (Figure 1A). The expression of the *Stim1L* isoform, responsible for rapid SOCE activation and extracellular Ca^2+^ entry in skeletal muscle and brain [19], was reduced to a similar extent (suppl. Figure 1F). Western blot confirmed the decrease of overall STIM1 protein levels in *Stim1* ^*+/−*^ muscle (Figure 1B). Of note, STIM1 mRNA and protein levels increased between 4 and 18 months in WT and *Stim1* ^*+/−*^ muscle, possibly reflecting an adaptation of SOCE activity to ageing. Altogether, these data show that STIM1 reduction has no major pathogenic effect on embryonic or postnatal development in mice and does not significantly interfere with life expectancy.

To correlate reduced STIM1 expression with muscle physiology, we performed a series of Ca^2+^ measurements on primary myoblasts differentiated into myotubes. Compared with controls, basal Ca^2+^ levels were decreased by 16% in *Stim1* ^*+/−*^ myotubes (Figure 1C). However, thapsigargin‐induced depletion of the reticular Ca^2+^ stores and subsequent addition of 5 mM Ca^2+^ to the medium did not reveal any differences in extracellular Ca^2+^ entry between WT and *Stim1* ^*+/−*^ myotubes (Figure 1D). This indicates that the reduction of STIM1 expression had minor but measurable effects on SOCE fine tuning, while maximal SOCE activation remained functional.

**FIGURE 2:**
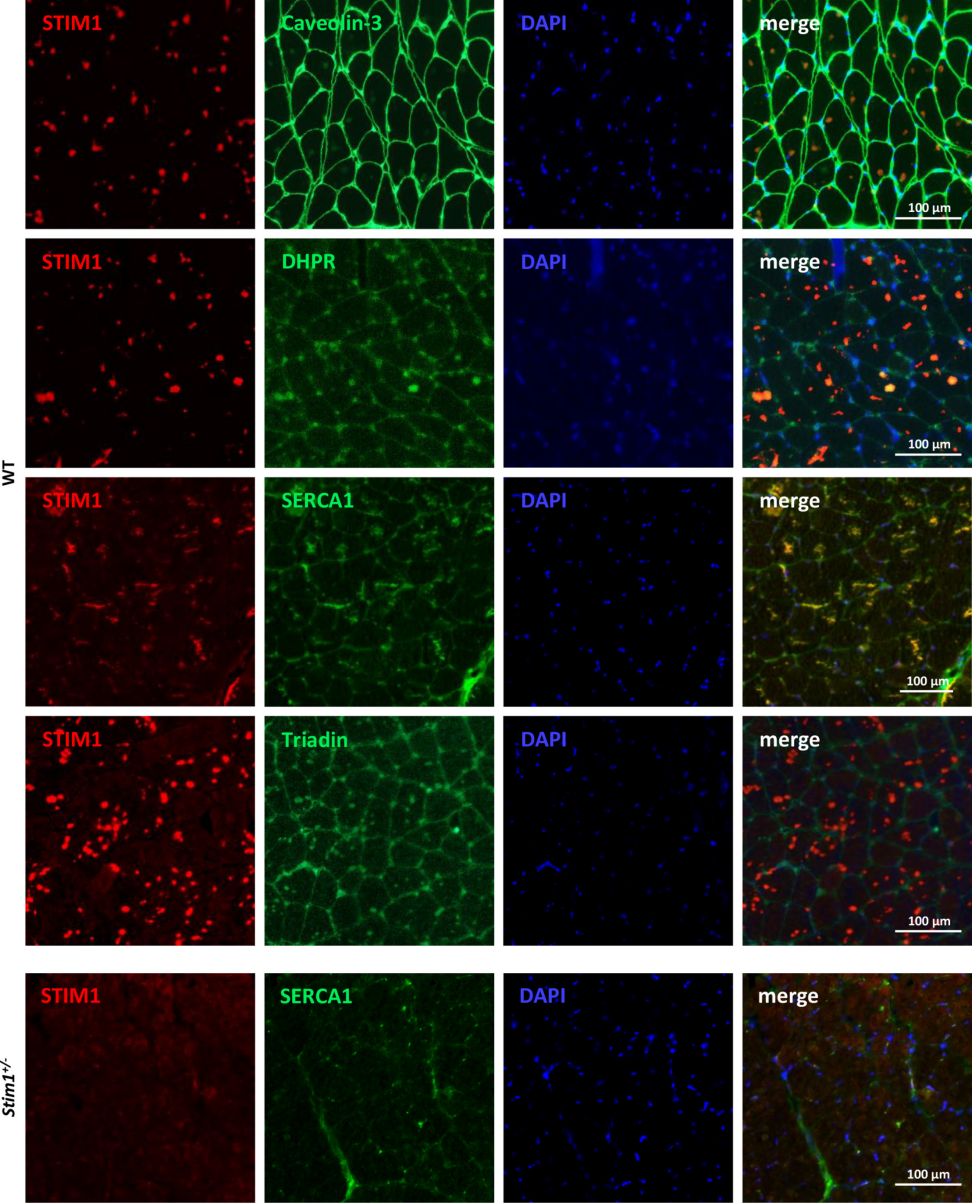
Tubular aggregate composition in WT mice at 18 months Immunofluorescence studies on transverse muscle sections from 18‐month‐old WT male mice detected STIM1, Caveolin‐3, DHPR, SERCA1 and Triadin within the tubular aggregates. Nuclei stained blue with DAPI. No tubular aggregates were detectable in *Stim1* ^*+/−*^ males at 18 months.

Histological and ultrastructural examination of tibialis anterior sections from 18‐month‐old animals revealed the presence of tubular aggregates only in WT male mice. In contrast, aggregates were undetectable by light and electron microscopy in WT females and in both *Stim1* ^*+/−*^ male and female mice, highlighting a critical role of STIM1 in tubular aggregate formation (Figure 1E). To investigate tubular aggregate composition in aged WT male mice, we performed immunofluorescence experiments on muscle sections. In line with previous studies on muscle biopsies from TAM/STRMK patients, we detected the ER proteins STIM1, SERCA1 and TRDN within the aggregates (Figure 2). In addition, we also found faint positive signals for the plasma membrane proteins Caveolin‐3 and DHPR, accrediting previous studies reporting the presence of DHPR in tubular aggregates in patients with congenital myasthenic syndrome (CMS) [20] and indicating that tubular aggregates are not solely composed of SR proteins. Overall, these findings illustrate that tubular aggregates in aged male rodents are STIM1‐dependent, have a similar composition as in TAM/STRMK patients and are suitable models to study aggregate formation in disease and age‐related processes.

### 3.2 STIM1 Reduction Impairs Late‐Stage Muscle Function

To understand the impact of the presence or absence of tubular aggregate on muscle functionality, we next assessed muscle force, contraction and relaxation in *Stim1* ^*+/−*^ and WT male mice.

**FIGURE 3:**
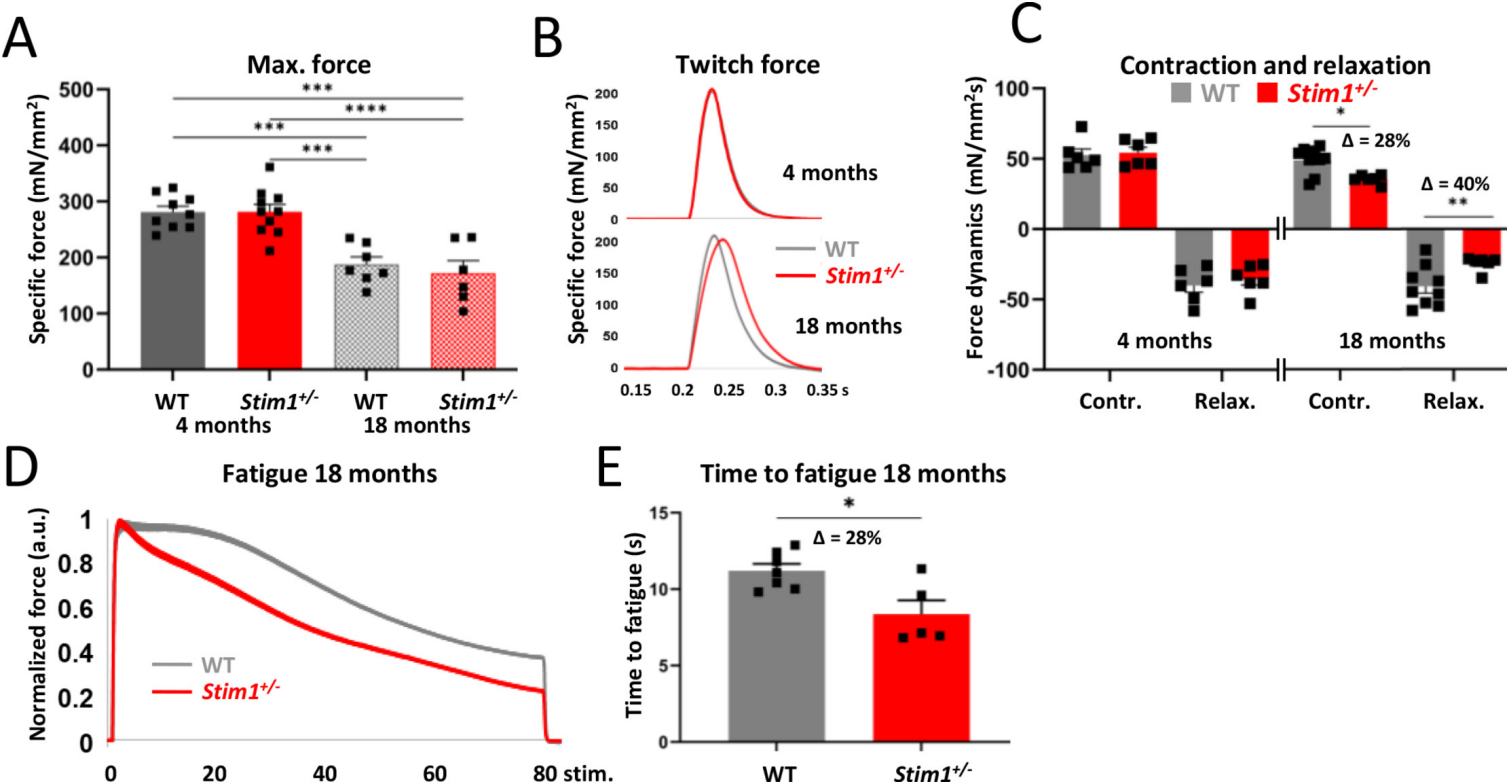
Altered muscle function in aged *Stim1* ^*+/−*^ males. (A) Maximal muscle force was equivalent in WT and *Stim1* ^*+/−*^ tibialis anterior at 4 and 18 months of age. (B) Representative graphs of overlapping muscle twitch curves of WT and *Stim1* ^*+/−*^ tibialis anterior at 4 month and of dephased curves at 18 months. (C) Tibialis anterior contraction and relaxation were similar in WT and *Stim1* ^*+/−*^ mice at 4 month and delayed in *Stim1* ^*+/−*^ mice at 18 months. (D) Representative graph of diverging fatigues curves in WT and *Stim1* ^*+/−*^ male mice at 18 months. (E) Quantification evidenced enhanced fatigue of *Stim1* ^*+/−*^ tibialis anterior compared with WT controls at 18 months. Data are presented as mean values ± SEM. For the comparison of two groups, the unpaired Student's *t* test with or without Welch's correction was applied, and for the comparison of more than two groups, data were analyzed with one‐way ANOVA followed by Tukey's multiple comparison test. Significant differences are indicated as *p < 0.05, **p < 0.01, ***p < 0.001 and ****p < 0.0001.

At 4 months, grip strength and hanging time were equivalent in WT and *Stim1* ^*+/−*^ mice (suppl. Figure 2A,B), and grip force was also comparable in WT and *Stim1* ^*+/−*^ littermates at 18 months (suppl. Figure 2D). In situ experiments on anaesthetized animals did not reveal differences in maximal force and contraction/relaxation dynamics at 4 months (Figure 3A–C). However, although maximal force remained similar between WT and *Stim1* ^*+/−*^ cohorts at 18 months (Figure 3A), the in situ muscle force parameters significantly diverged. Indeed, *Stim1* ^*+/−*^ mice showed a dephased tibialis anterior twitch curve with a delay in both muscle contraction (*Δ* = 28%) and relaxation (*Δ* = 40%) kinetics (Figure 3B,C). Moreover, the fatigue curves of 18‐month‐old WT and *Stim1* ^*+/−*^ mice differed in shape with faster fatigue in *Stim1* ^*+/−*^ mice compared with WT littermates, while no disparity in the time to fatigue was noticeable at 4 months (Figure 3D and suppl. Figure 2C). To establish a time course and refine the interval in which muscle function in WT and *Stim1* ^*+/−*^ mice disperse, we examined mouse cohorts of intermediate age. At 10 months, maximal force as well as muscle contraction and relaxation kinetics of WT and *Stim1* ^*+/−*^ mice was indistinguishable (suppl. Figure 3A,B), while *Stim1* ^*+/−*^ mice showed a nonsignificant tendency of increased fatigability (suppl. Figure 3C,D). Tubular aggregates were exceedingly rare and barely detectable in WT tibialis anterior (suppl. Figure 3E) at 10 months. Taken together, STIM1 reduction has no discernible effect on muscle function in young adult male mice but compromises muscle contraction/relaxation and resistance to fatigue in aged rodents.

### 3.3 STIM1 Reduction Induces Fibre Type Shift and Mitochondrial Dysfunction in Aged Mice

**FIGURE 4:**
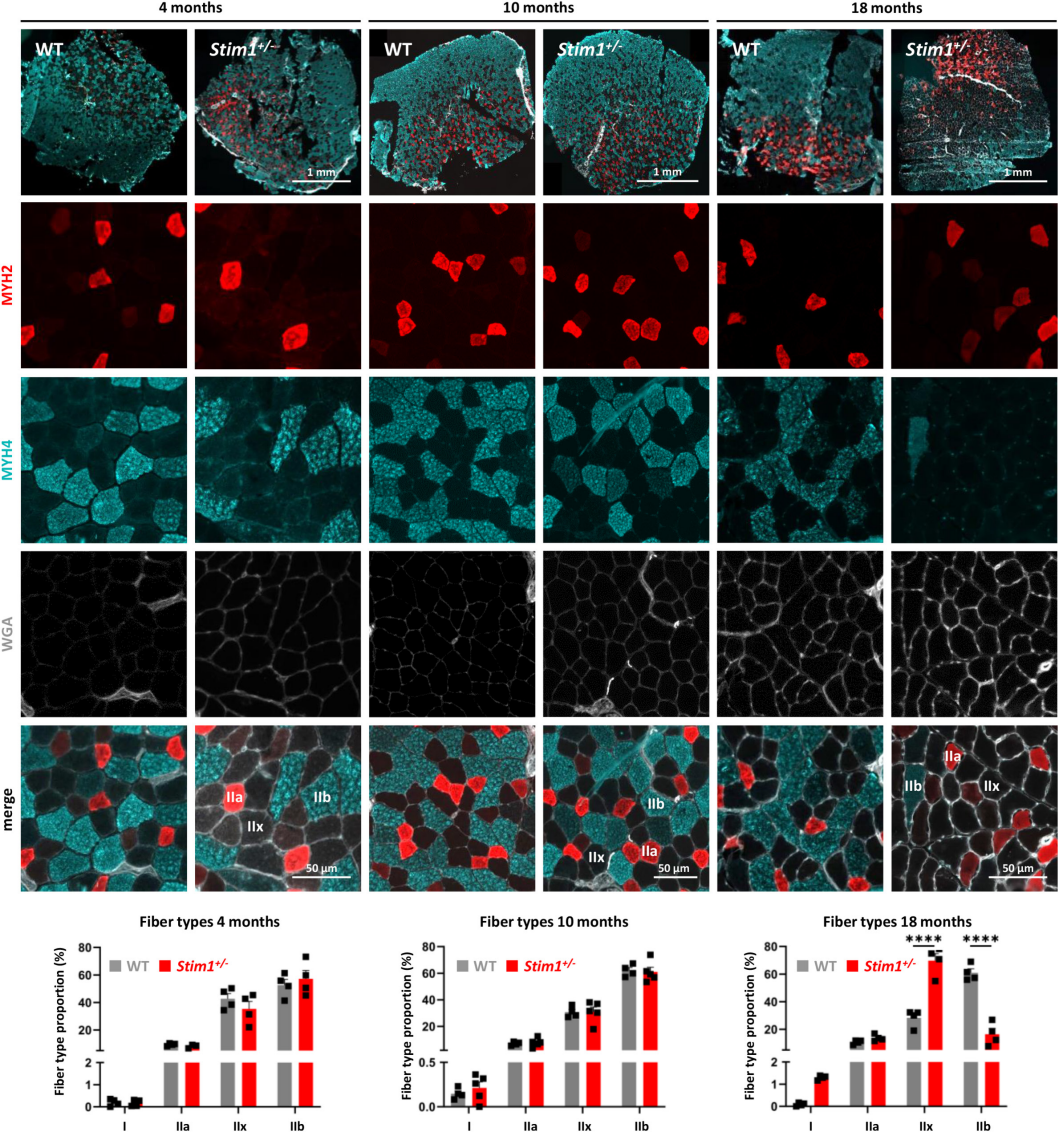
Age‐related fibre type shift in *Stim1* ^*+/−*^ tibialis anterior. Immunostaining of transverse tibialis anterior sections with specific anti‐myosin heavy chain (MYH) antibodies and quantification of the fluorescence intensities revealed normal fibre type proportion in *Stim1* ^*+/−*^ males at 4 and 10 months. At 18 months, the ratio of type I and IIx fibres was increased and the ratio of type IIb fibres decreased in *Stim1* ^*+/−*^ muscle compared with WT controls. Data are presented as mean values ± SEM. *T* test with Welch's correction. Significant differences are indicated as ****p < 0.0001.

To explore the mechanisms leading to muscle dysfunction in aged *Stim1* ^*+/−*^ males, we next characterized fibre‐type composition in tibialis anterior. Immunofluorescence experiments using antibodies for fibre type–specific myosin heavy chains MYH2 and MYH4 revealed a partial shift of fibre type proportion in WT muscle with age. *Stim1* ^*+/−*^ mice exhibited a normal ratio and distribution of type IIa, IIx and IIb myofibres at 4 and 10 months (Figure 4). At 18 months, *Stim1* ^*+/−*^ mice displayed a significantly higher ratio of type I and type IIx fibres (+41%) and a decreased proportion of type IIb fibres (−45%) compared with WT tibialis anterior.

Oxidative type I, mixed type IIa and glycolytic type IIx and IIb muscle fibres differ in mitochondrial content and the primary metabolic pathway to produce force [21]. Moreover, mitochondrial function and homeostasis decays with age and has been associated with increasing fatigability, a major phenotype in 18‐month‐old *Stim1* ^*+/−*^ mice. To investigate whether the apparent age‐related shift of *Stim1* ^*+/−*^ tibialis anterior towards slower myofibre types may be concomitant with mitochondrial modifications, we assessed mitochondrial distribution and activity in WT and *Stim1* ^*+/−*^ muscle at 18 months.

**FIGURE 5:**
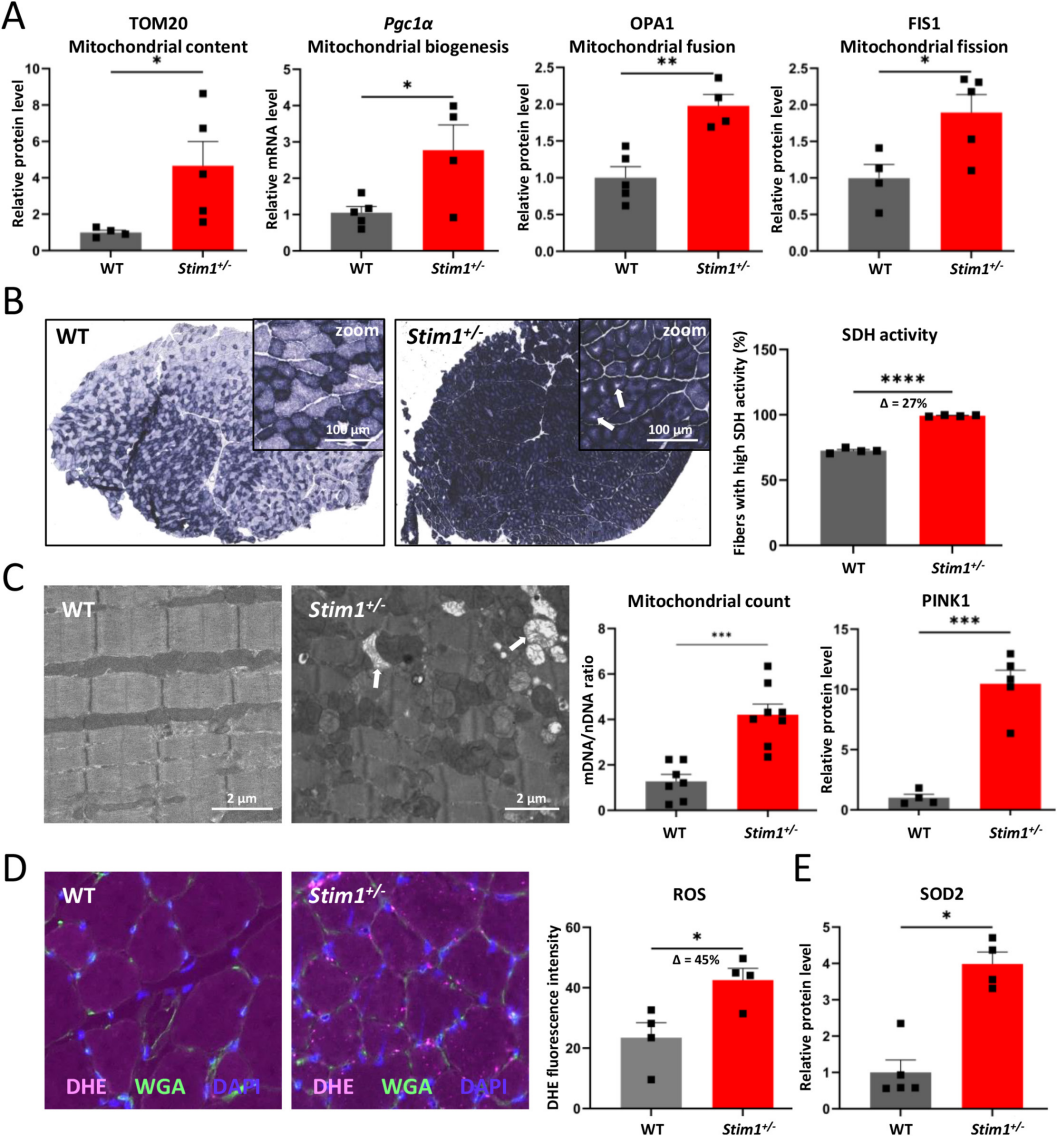
Altered mitochondrial distribution and function in aged *Stim1* ^*+/−*^ males. (A) Increased mRNA or protein levels of biomarkers for mitochondrial quantity (TOM20), mitochondrial biogenesis (*Pgc1α*), mitochondrial fusion (OPA1) and mitochondrial fission (FIS1) in *Stim1* ^*+/−*^ muscle samples compared with WT controls. (B) SDH (succinate dehydrogenase) staining of transverse tibialis anterior sections revealed more pronounced signal intensities in *Stim1* ^*+/−*^ mice than in WT littermates. Insets reflect higher magnifications and illustrate the absence of dark SDH signals in central myofibre areas in *Stim1* ^*+/−*^ mice. (C) Electron microscopy supported mitochondrial abundance in *Stim1* ^*+/−*^ tibialis anterior and detected structural anomalies indicative of mitophagy (arrows). Quantititaive PCR revealed an increased ratio of mitochondrial versus nuclear DNA in *Stim1* ^*+/−*^ mice, and quantification of the biomarker PINK1 confirmed increased mitophagy in *Stim1* ^*+/−*^ muscle. (D) DHE (dihydroethidium) tests on transverse tibialis anterior sections uncovered considerably more reactive oxygen species (ROS, pink signals) in *Stim1* ^*+/−*^ muscle compared with WT controls. Nuclei stained blue with DAPI, and plasma membranes green with WGA (E) Protein levels of the mitochondrial superoxide dismutase SOD2 were increased in *Stim1* ^*+/−*^ mice compared with WT littermates. Data are presented as mean values ± SEM. *T* test with Welch's correction. Significant differences are indicated as *p < 0.05, **p < 0.01, ***p < 0.001 and ****p < 0.0001.

First, we analyzed diverse markers of mitochondrial quantity and dynamics. Compared with WT controls, *Stim1* ^*+/−*^ muscle samples exhibited significantly elevated mRNA or protein levels of TOM20, *Pgc1α*, OPA1 and FIS, indicative of enhanced mitochondrial biogenesis, fusion and fission, respectively (Figure 5A and suppl. Figure 4A). This was in accordance with SDH immunohistochemistry data showing a 27% increase of mitochondrial activity in *Stim1* ^*+/−*^ muscle compared with WT controls (Figure 5B). Of note, higher magnifications evidenced irregular SDH signal intensities with central areas devoid of mitochondrial activity in *Stim1* ^*+/−*^ muscle. Ultrastructural investigations corroborated mitochondrial proliferation in *Stim1* ^*+/−*^ tibialis anterior, and quantitative PCR confirmed an increased ratio of mitochondrial DNA over nuclear DNA in *Stim1* ^*+/−*^ mice compared with WT littermates (Figure 5C). We also detected signs of mitophagy on electron microscopy sustained by increased protein levels of the mitophagy marker PINK1 (Figure 5C and suppl. Figure 4A).

Given the central role of mitochondria in the production of ROS, we conducted DHE tests to quantify free radicals in tibialis anterior and detected an almost twofold higher ROS intensity in *Stim1* ^*+/−*^ muscle compared with WT controls (Figure 5D). Furthermore, we noted increased protein levels of the mitochondrial superoxide dismutase SOD2, responsible for ROS neutralization, but not of the cytoplasmic SOD1, suggesting insufficient cytosolic ROS clearance (Figure 5E and suppl. Figure 4B). We also found quantitative alterations of the mitochondrial OXPHOS (oxidative phosphorylation) complex and especially increased levels of proteins forming complexes I and V, potentially accounting for the ROS overproduction in *Stim1* ^*+/−*^ muscle (suppl. Figure 4C). Taken together, these data support the idea that STIM1 reduction engenders mitochondrial dysregulation and dysfunction with rising age, which causes enhanced ROS production and ultimately leads to muscle fatigability.

### 3.4 STIM1 Reduction Essentially Impacts on Fast‐Twitch Muscle Fibres

All experiments on tubular aggregate formation, fibre type ratio, mitochondrial distribution and mitochondrial activity were performed on tibialis anterior, a fast‐twitch muscle essentially composed of type II myofibres. To extend our study and conclude on the impact of STIM1 reduction on other muscles with different fibre type composition, we examined the slow‐twitch soleus muscle, predominantly constituted of type I and type IIa fibres, and the gastrocnemius muscle, a mixed‐type muscle containing all types of fibres.

**FIGURE 6:**
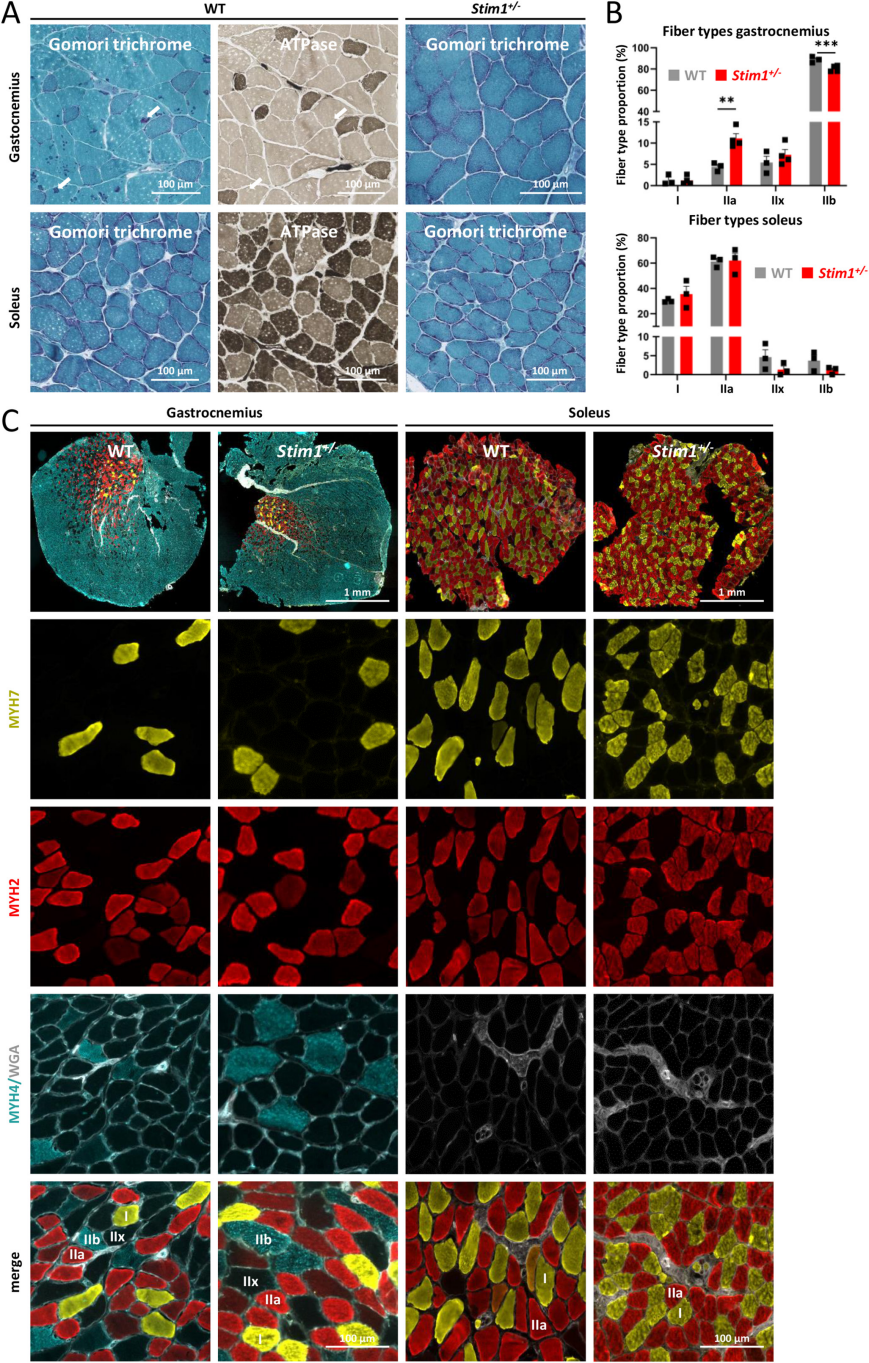
No major impact of STIM1 reduction on slow‐twitch muscle structure. (A) Histological analyses of serial muscle sections evidenced the presence of tubular aggregates only in type II myofibres in WT gastrocnemius muscle (arrows). No tubular aggregates were detected in WT soleus and in *Stim1* ^*+/−*^ gastrocnemius and soleus. (B–C) Immunofluorescence and quantification of signal intensities revealed comparable fibre type proportions in WT and *Stim1* ^*+/−*^ soleus, while *Stim1* ^*+/−*^ gastrocnemius manifested a shift in fibre type ratios compared with WT littermates. Data are presented as mean values ± SEM. *T* test with Welch's correction. Significant differences are indicated as **p < 0.01 and ***p < 0.001.

At 18 months, Gomori trichrome staining revealed the robust presence of tubular aggregates in the gastrocnemius muscle of WT mice, and serial sections stained with ATPase demonstrated that the aggregates were exclusively found in fast‐twitch type II myofibres (Figure 6A). Tubular aggregates were absent in the slow‐twitch soleus muscle and were also undetectable in *Stim1* ^*+/−*^ gastrocnemius and soleus. To better discern and quantify the individual fibre types, we performed immunofluorescence experiments on transverse muscle sections (Figure 6B,C). While the fibre type ratio was similar in WT and *Stim1* ^*+/−*^ soleus muscles, we noticed a shift in fibre type proportion in *Stim1* ^*+/−*^ gastrocnemius with an increase of type IIa myofibres and a decrease of type IIb myofibres compared with WT mice. Altogether, these findings largely confirmed our findings on tibialis anterior, and substantiated the link between STIM1 reduction, tubular aggregate formation and modifications of fibre type composition.

**FIGURE 7:**
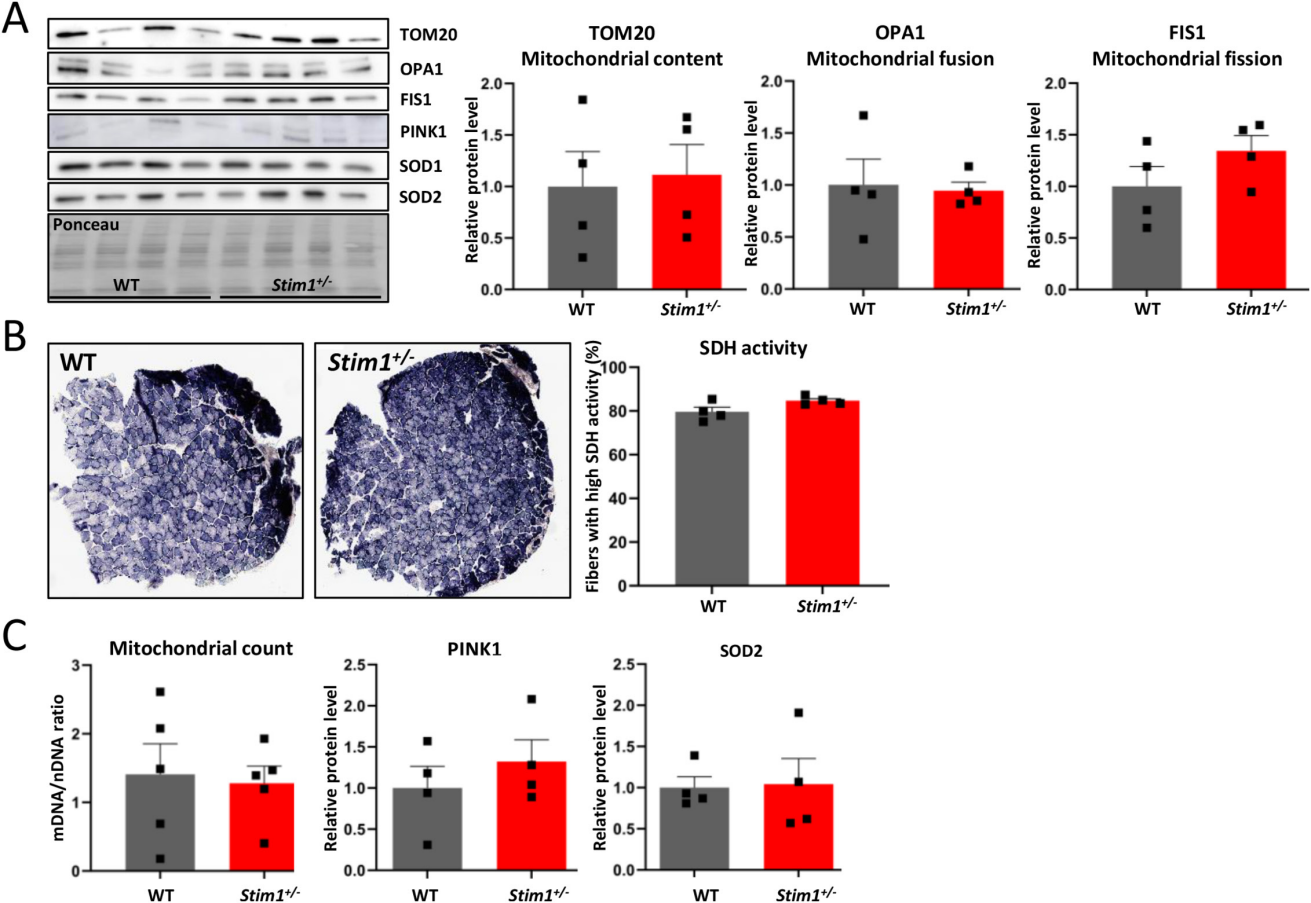
No major impact of STIM1 reduction on mitochondrial function in slow‐twitch myofibres. (A) Western blot and quantification of mitochondrial biomarkers did not reveal differences in mitochondrial numbers, fusion and fission between WT and *Stim1* ^*+/−*^ soleus at 18 months. (B) Comparable SDH intensities on transverse soleus sections from WT and *Stim1* ^*+/−*^ mice at 18 months. (C‐D) Similar mitochondrial numbers and protein levels of the mitophagy biomarker PINK1 and the mitochondrial superoxide dismutase SOD2 in WT and *Stim1* ^*+/−*^ mice at 18 months. Data are presented as mean values ± SEM. *T* test with Welch's correction.

To evaluate mitochondrial function in slow‐twitch muscle fibres, we analyzed soleus sections with the same set of experiments as for tibialis anterior. The protein levels of TOM20, OPA1 and FIS1, respectively, serving as biomarkers for mitochondrial quantity, fusion and fission were comparable in WT and in *Stim1* ^*+/−*^ mice (Figure 7A), and we did not observe differences in SDH signal intensities on immunohistochemically stained muscle sections (Figure 7B). Moreover, PINK1 and SOD2 protein levels were normal in *Stim1* ^*+/−*^ soleus, indicating the absence of profuse mitophagy or ROS production (Figure 7C). In conclusion, STIM1 reduction appears to have no or only minor pathogenic effects on slow‐twitch muscle fibres.

## 4 Discussion

Sarcopenia is defined as age‐related decrease in lean muscle mass and occurs in conjunction with loss of strength and physical performance. In male mice, sarcopenia goes along with the formation of tubular aggregates, which are central or subsarcolemmal basophilic inclusions primarily found in fast‐twitch type II muscle fibres [14]. Tubular aggregates were also described in diverse human neuromuscular disorders such as myasthenic syndrome, hypokalemic periodic paralysis (hypoPP), inflammatory or ethyltoxic myopathies and represent the main histopathological hallmark in muscle biopsies from TAM/STRMK patients [1, 9, 11, 12]. TAM/STRMK is caused by dominant STIM1 mutations leading to disturbances of intracellular Ca^2+^ balance [5]. However, the relevance of tubular aggregates and their potential contribution to disease and age‐related decline in muscle force is currently not understood. The present work addressed the physiopathological link between tubular aggregates, ageing, STIM1 and Ca^2+^, and we provide morphological, physiological and functional data.

### 4.1 A Critical Role of STIM1 in Maintenance of Muscle Physiology

STIM1 is an essential component of the SOCE mechanism and acts as an elemental regulator of Ca^2+^ homeostasis. STIM1 gain‐of‐function mutations induce SOCE overactivity and give rise to TAM/STRMK [5], while loss of STIM1 accompanied by SOCE failure impairs extracellular Ca^2+^ entry and causes severe combined immunodeficiency (SCID), characterized by chronic infections, autoimmunity, muscular hypotonia and ectodermal dysplasia [22, 23]. Heterozygous carriers of STIM1 loss‐of‐function mutations are described as healthy, illustrating that the remaining STIM1 expression or functionality of 50% is sufficient to preserve vital SOCE activity in immune cells, myofibres, ameloblasts and other cell types. In accordance, *Stim1* ^*+/−*^ mice were born with the expected Mendelian ratio, were fertile and showed unremarkable postnatal development with normal body and organ weight as well as average life expectancy.

However, at 18 months of age, *Stim1* ^*+/−*^ muscles displayed an altered fibre type composition, enhanced mitochondrial proliferation associated with increased ROS production, decelerated muscle contraction and relaxation, and exacerbated fatigue. All are classical signs of sarcopenia and none were detectable at 4 months of age, indicating that the deficit of STIM1 is more efficiently compensated at a younger age or that insufficient SOCE entails rising dysregulations of Ca^2+^‐dependent signalling pathways, which lead to a progressive decrease in muscle performances. This is of particular medical importance for carriers of STIM1 loss‐of‐function mutations, who may bear a higher risk of developing sarcopenia and a decline in muscle force in later years. It also highlights the direct role of STIM1 in the maintenance of muscle physiology and its ability to partially prevent age‐related deterioration of muscle function. This is supported by the continuously increasing STIM1 levels in ageing WT mice, suggesting that the regulation of STIM1 expression is a way to adjust SOCE and Ca^2+^ balance to metabolic modifications during the ageing process.

In mice, skeletal muscle is composed of slow‐twitch type I and fast‐twitch type IIa, IIx and IIb fibres, and the ratio and distribution of fibre types reflect an adaptation of specific muscle groups to a range of activities requiring endurance, fast power or a mix of both [24, 25]. In 18‐month‐old *Stim1* ^*+/−*^ mice, we observed a shift of type IIb to type IIx and I fibres in tibialis anterior and an increased proportion of type IIa fibres in gastrocnemius, indicating an overall tendency of a conversion from faster to slower myofibres. A similar relative reduction of powerful type II muscle fibres is also seen in humans and possibly contributes to the decrement of general muscle strength with age [26]. The individual fibre types differ in their reticular Ca^2+^ content, Ca^2+^ sensitivity and resilience to oxidative stress [27, 28]. It is therefore possible that the changing fibre type composition in aged *Stim1* ^*+/−*^ muscle directly arises from decreased resting Ca^2+^ levels affecting multiple Ca^2+^‐dependent cellular processes. Alternatively, the transition towards slower and mitochondrial‐rich myofibres may also represent a compensatory effect and the attempt of the muscles to counteract the fatigability of *Stim1* ^*+/−*^ muscles through a switch from glycolytic to oxidative energy systems.

### 4.2 A Potentially Protective Effect of Tubular Aggregates

One of the striking morphological features of aged *Stim1* ^*+/−*^ males is the absence of tubular aggregates on muscle sections, and the same observation was made in mice with muscle‐specific depletion of ORAI1 [29]. In analogy to *Stim1* ^*+/−*^ mice, *cOrai1* ^*−/−*^ mice manifested progressive muscle force decrease, exercise intolerance and mitochondrial damage. Although both murine models diverge in several aspects and notably in reduced body mass, shortened life span and the appearance of premature ageing including faded fur and eye opacity in *cOrai1* ^*−/−*^ mice, both pinpoint the correlation between absent tubular aggregates and increasing deficits in muscle force and exercise tolerance with age. This underscores the relevance of SOCE in the preservation of muscle physiology and its direct implication in tubular aggregate formation.

Tubular aggregates stain red with Gomori trichrome and dark blue with NADH‐TR (nicotinamide adenine dinucleotide‐tetrazolium reductase staining) but are nonreactive to SDH (succinyl dehydrogenase) or COX (cytochrome oxidase) histochemistry, indicating that they are not of mitochondrial origin [9, 11]. At higher magnification, tubular aggregates appear as regular arrays of single‐ or double‐walled membrane tubules with diameters ranging from 20 to 200 nm [9, 30]. They contain various sarcoplasmic reticulum proteins including STIM1, the SERCA1 and SERCA2 Ca^2+^ pumps, the Ca^2+^ release channel RyR1 or the Ca^2+^ buffer calsequestrin [5, 6, 9, 31, 32, 33], strongly suggesting that they originate from the SR. Our immunofluorescence experiments also evidenced the presence of plasma membrane proteins within the aggregates, indicating that at least late‐stage tubular aggregates can sequester and incorporate membrane‐bound proteins or vesicles transporting membrane proteins across the cell.

Considering that tubular aggregates also contain large amounts of Ca^2+^ [9, 34], it is possible that they serve as a trap to seclude excessive Ca^2+^ and exert a protective role by reducing cellular stress and preventing myofibre breakdown. Indeed, *Stim1* ^*D84G/+*^, *Stim1* ^*I115F/+*^ and *Stim1* ^*R304W/+*^ mice harbouring different STIM1 gain‐of‐function mutations do not feature tubular aggregates on muscle samples, but manifest significantly more dystrophic signs of muscle fibre degeneration compared with TAM/STRMK patients and *Orai1* ^*G98S/+*^/*Orai1* ^*V109M/+*^ mice, all exhibiting abundant tubular aggregates in myofibres [35, 36, 37, 38, 39].

Nevertheless, it should be noted that tubular aggregates are generally absent in female WT mice without a concurrent increase in muscle fibre degeneration and that the prevalence of tubular aggregates is reduced in male WT mice undergoing continuous exercise or treated with oestrogen or antioxidants [40, 41]. Moreover, a possible age‐related occurrence of tubular aggregates in humans has barely been studied, and in TAM/STRMK patients, tubular aggregates are predominantly found in type II myofibres in both males and females but can also occur in type I fibres [2]. These points suggest that tubular aggregate formation is a multifactorial process influenced by genetic background, hormones, physical activity, nutrition and possibly other environmental modifiers and that the absence or presence of tubular aggregates is not necessarily a suitable parameter to determine health or disease status. The absence of tubular aggregates in outbred mouse strains and in inbred female mice also suggests that the natural and gender‐independent loss of muscle mass with age occurs independently of aggregate formation. This is supported by the comparable body weight and muscle force of WT and *Stim1* ^*+/−*^ littermates at 18 months. However, the aberrant muscle function and morphology in elder *Stim1* ^*+/−*^ males bereft of tubular aggregates in fast‐twitch myofibres suggest the existence of a correlation between the presence/absence of tubular aggregates and the preservation/deterioration of muscle functionality.

## 5 Concluding Remarks

*Stim1* ^*+/−*^ mice manifested signs of accelerated ageing including deferred muscle contraction, exercise intolerance, mitochondrial dysfunction, increased oxidative stress and—as the principal histological hallmark on muscle section—the absence of tubular aggregates. The present study evidenced a direct role of STIM1 and intracellular Ca^2+^ handling in tubular aggregate formation and the maintenance of muscle physiology and supports the idea that tubular aggregates may have a protective role to partially prevent age‐related muscle deterioration.

## Funding

This work of the Interdisciplinary Thematic Institute IMCBio+, as part of the ITI 2021–2028 program of the University of Strasbourg, CNRS and Inserm, was supported by IdEx Unistra (ANR‐10‐IDEX‐0002), SFRI‐STRAT'US (ANR‐20‐SFRI‐0012), EUR IMCBio (ANR‐17‐EURE‐0023) under the framework of the France 2030 Program, *Association Française contre les Myopathies* (AFM‐Téléthon 22734 and 23933) and *Fondation pour la Recherche Médicale* (FRM, PLP20170939073 to Roberto Silva‐Rojas).

## Ethics Statement

Animal care and experimentation were in accordance with French and European guidelines and approved by the institutional ethics committee Com'eth (Project Numbers APAFIS #2016031110589922, #202052516535988 and #2019062813376603) and accredited by the French Ministry for Superior Education and Research and in accordance with the Directive of the European Parliament (2010/63/EU).

## Conflicts of Interest

RSR, JL and JB declare the following patent: EP21306473.6—method for treating tubular aggregate myopathy and Stormorken syndrome.

## Supporting information


**Figure S1:** **No impact of STIM1 reduction on body mass, organ mass and survival.** (A) Strategy of *Stim1* exon 2 deletion via the Cre‐LoxP recombination system. (B) *Stim1* ^*+/−*^ mice were born with expected Mendelian ratio. (C–E) Comparable body weight, body length, organ weight and survival rates of WT and *Stim1* ^*+/−*^ mice until 18 months. (F) mRNA levels of both *Stim1* and *Stim1L* isoforms were reduced in *Stim1* ^*+/−*^ mice. Data are presented as mean values ± SEM. *T* test with Welch's correction. Significant differences are indicated as *p < 0.05.
**Figure S2: Normal general muscle force of Stim1** ^***+/−***^ **males at 4 and 18 months.** (A–B) Equivalent grip force and hanging time capacities upside down a cage grid of WT and *Stim1* ^*+/−*^ mice at 4 months. (C) Quantification of the time to reach 50% muscle force showed comparable fatigue of WT and *Stim1* ^*+/−*^ tibialis anterior at 4 months. (D) Comparable grip strength of WT and *Stim1* ^*+/−*^ mice at 18 months. Data are presented as mean values ± SEM. *T* test with Welch's correction.
**Figure S3: Normal muscle function in 10‐month‐old Stim1** ^***+/−***^ **males**. (A–D) At 10 months, WT and *Stim1* ^*+/−*^ mice manifested comparable maximal muscle force, muscle contraction and relaxation kinetics as well as fatigue. (E) Tubular aggregates are scarce in WT tibialis anterior sections stained with Gomori trichrome (arrow) and absent in *Stim1* ^*+/−*^ mice.
**Figure S4: Abnormal mitochondrial biomarkers in Stim1** ^***+/−***^ **mice at 18 months.** (A–B) Western blots illustrating abnormal protein levels of diverse mitochondrial biomarkers in *Stim1* ^*+/−*^ tibialis anterior at 18 months. Protein levels of the cytosolic superoxide dismutase SOD1 were comparable in WT and *Stim1* ^*+/−*^ mice. Ponceau served as loading control. (C) Western blot and quantification of OXPHOS proteins revealed increased protein levels of mitochondrial complexes I and V in *Stim1* ^*+/−*^ muscle compared with controls. Data are presented as mean values ± SEM. *T* test with Welch's correction. Significant differences are indicated as **p < 0.01 and ****p < 0.0001.

**Table S1:** List and sequences of genotyping and RT‐qPCR primers.
