# Supplementary figures and images for: STIM1 Reduction Prevents Tubular Aggregate Formation and Compromises Muscle Performance in Ageing Mice
Source: J Cachexia Sarcopenia Muscle. 2025 Dec 7;16(6):e70151. doi: 10.1002/jcsm.70151 (PMC12682393; doi:10.1002/jcsm.70151)

# Supplementary Figure 1

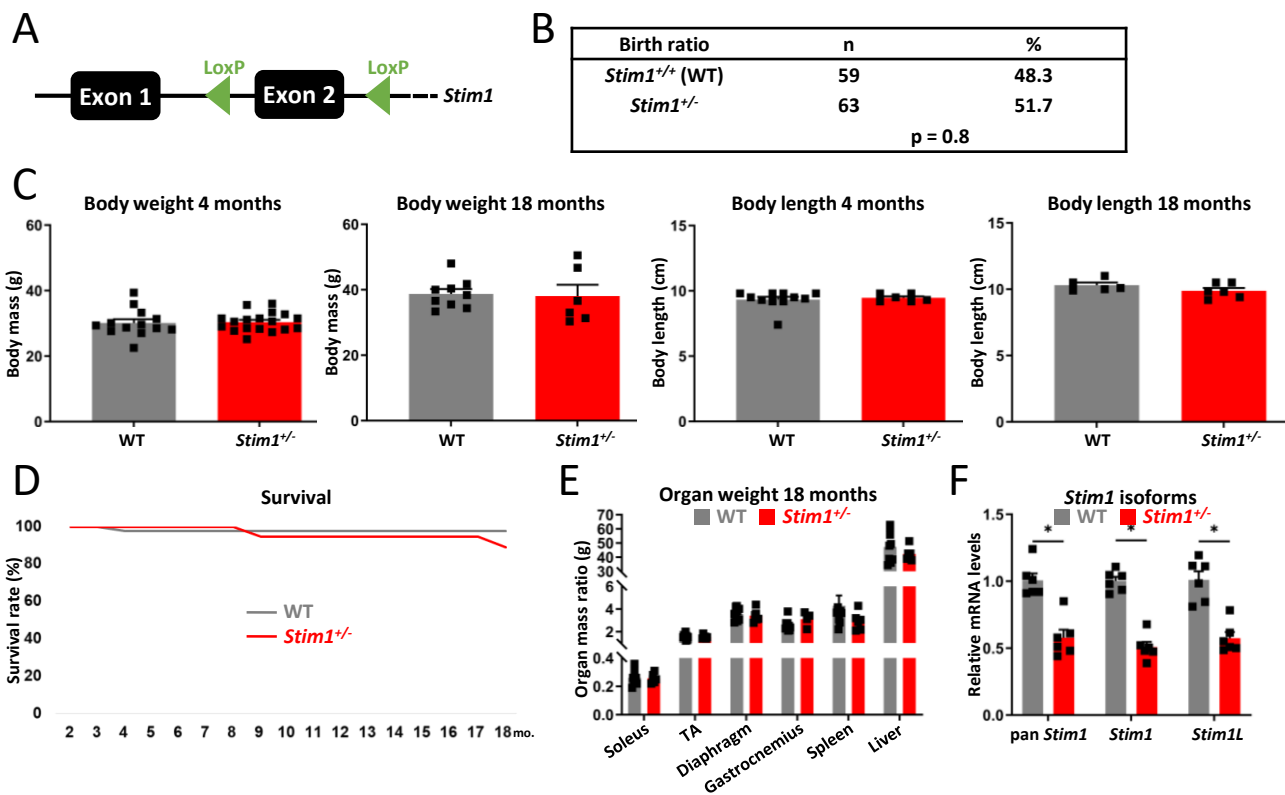

# Supplementary Figure 2

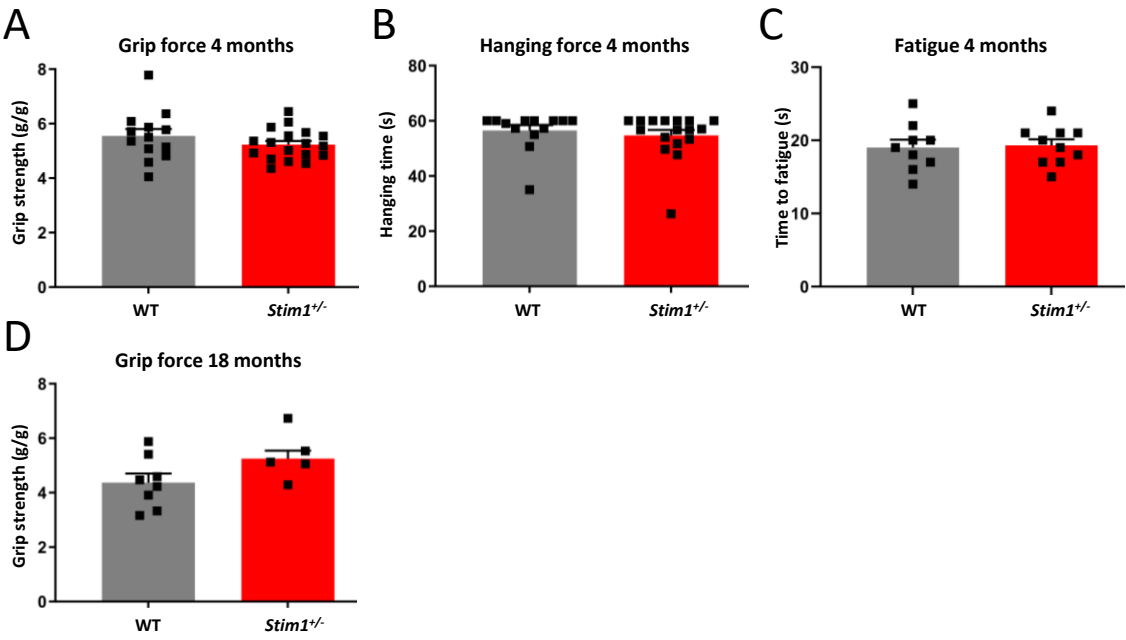

# Supplementary Figure 3

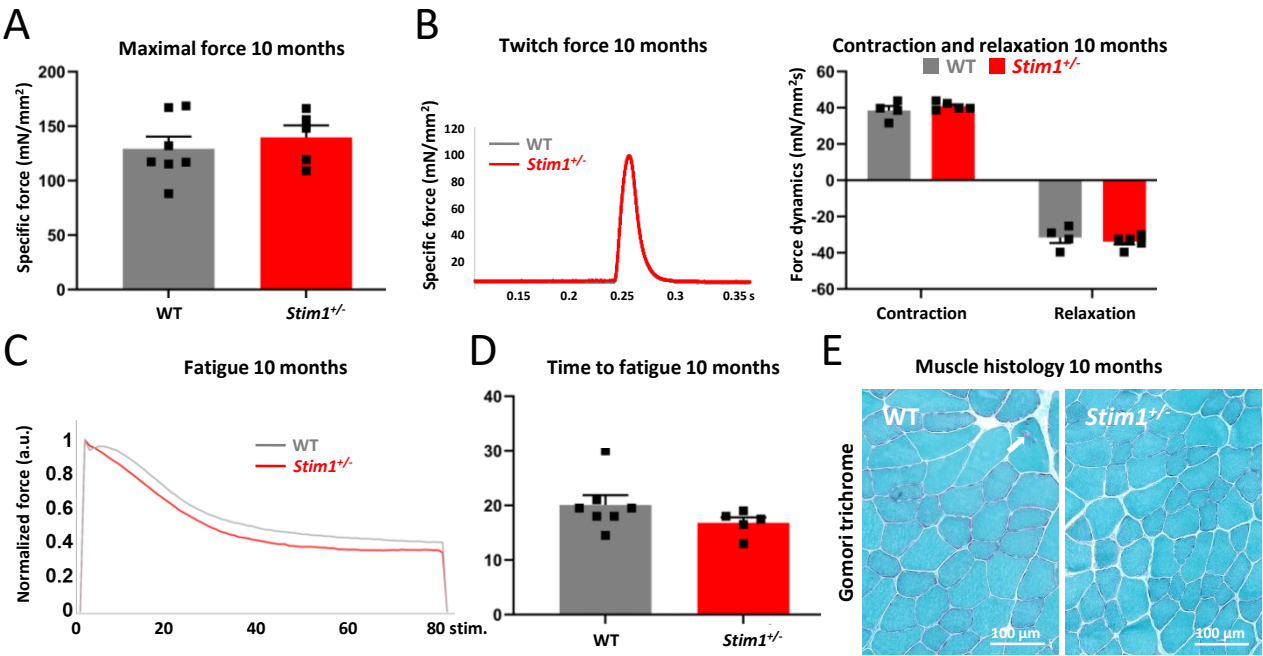

Supplementary Figure 4

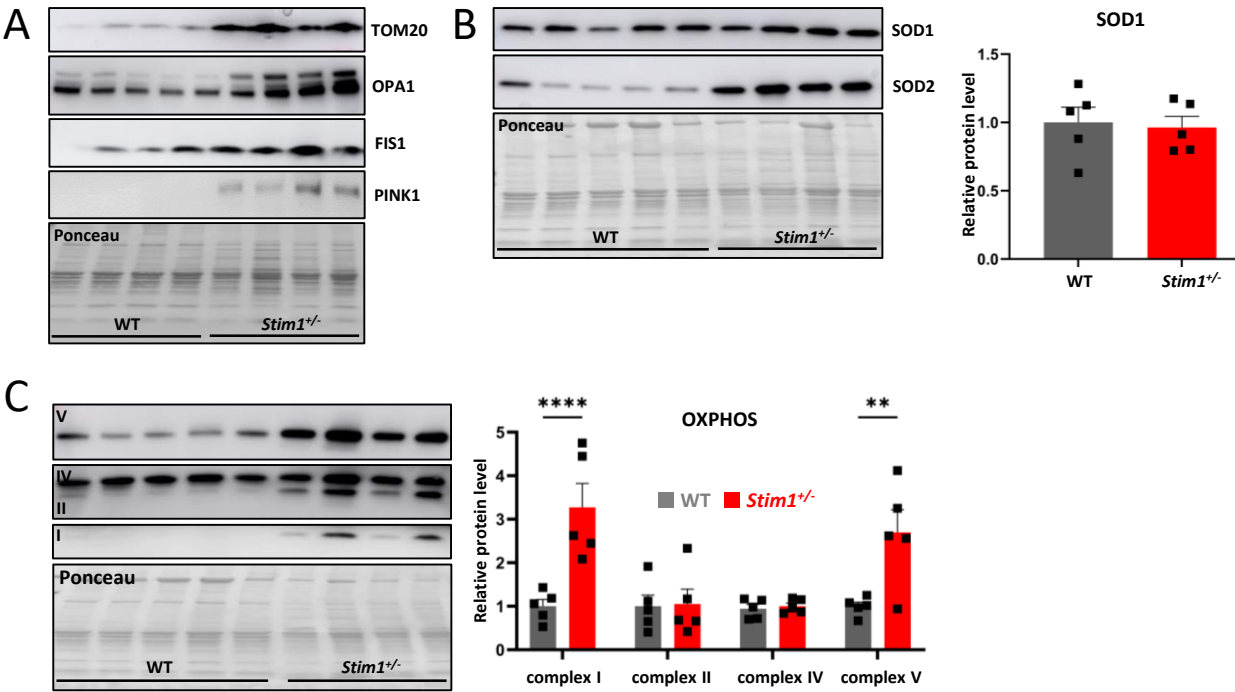

Supplement: Supplementary file 1 — Figure S1: No impact of STIM1 reduction on body mass, organ mass and survival. (A) Strategy of Stim1 exon 2 deletion via the Cre‐LoxP recombination system. (B) Stim1 +/− mice were born with expected Mendelian ratio. (C–E) Comparable body weight, body length, organ weight and survival rates of WT and Stim1 +/− mice until 18 months. (F) mRNA levels of both Stim1 and Stim1L isoforms were reduced in Stim1 +/− mice. Data are presented as mean values ± SEM. T test with Welch's correction. Significant differences are indicated as *p < 0.05. Figure S2: Normal general muscle force of Stim1 +/− males at 4 and 18 months. (A–B) Equivalent grip force and hanging time capacities upside down a cage grid of WT and Stim1 +/− mice at 4 months. (C) Quantification of the time to reach 50% muscle force showed comparable fatigue of WT and Stim1 +/− tibialis anterior at 4 months. (D) Comparable grip strength of WT and Stim1 +/− mice at 18 months. Data are presented as mean values ± SEM. T test with Welch's correction. Figure S3: Normal muscle function in 10‐month‐old Stim1 +/− males. (A–D) At 10 months, WT and Stim1 +/− mice manifested comparable maximal muscle force, muscle contraction and relaxation kinetics as well as fatigue. (E) Tubular aggregates are scarce in WT tibialis anterior sections stained with Gomori trichrome (arrow) and absent in Stim1 +/− mice. Figure S4: Abnormal mitochondrial biomarkers in Stim1 +/− mice at 18 months. (A–B) Western blots illustrating abnormal protein levels of diverse mitochondrial biomarkers in Stim1 +/− tibialis anterior at 18 months. Protein levels of the cytosolic superoxide dismutase SOD1 were comparable in WT and Stim1 +/− mice. Ponceau served as loading control. (C) Western blot and quantification of OXPHOS proteins revealed increased protein levels of mitochondrial complexes I and V in Stim1 +/− muscle compared with controls. Data are presented as mean values ± SEM. T test with Welch's correction. Significant differences are indicated as **p < [file JCSM-16-e70151-s002.pdf]
