# Supplementary material for: STIM1 Reduction Prevents Tubular Aggregate Formation and Compromises Muscle Performance in Ageing Mice
Source: J Cachexia Sarcopenia Muscle. 2025 Dec 7;16(6):e70151. doi: 10.1002/jcsm.70151 (PMC12682393; doi:10.1002/jcsm.70151)
Supplement: Supplementary file 2 — Table S1: List and sequences of genotyping and RT‐qPCR primers. [file JCSM-16-e70151-s001.docx]

**SUPPLEMENTARY MATERIAL**

**Supplementary figure 1. No impact of STIM1 reduction on body mass, organ mass, and survival.** (A) Strategy of *Stim1* exon 2 deletion via the Cre-LoxP recombination system. (B) *Stim1^+/-^* mice were born with expected Mendelian ratio. (C-E) Comparable body weight, body length, organ weight, and survival rates of WT and *Stim1^+/-^* mice until 18 months. (F) mRNA levels of both *Stim1* and *Stim1L* isoforms were reduced in *Stim1^+/-^* mice. Data are presented as mean values +/- SEM. T-test with Welch’s correction. Significant differences are indicated as *p<0.05.

**Supplementary figure 2. Normal general muscle force of *Stim1^+/-^* males at 4 and 18 months.** (A-B) Equivalent grip force and hanging time capacities upside down a cage grid of WT and *Stim1^+/-^* mice at 4 months. (C) Quantification of the time to reach 50% muscle force showed comparable fatigue of WT and *Stim1^+/-^* tibialis anterior at 4 months. (C) Comparable grip strength of WT and *Stim1^+/-^* mice at 18 months. Data are presented as mean values +/- SEM. T-test with Welch’s correction.

**Supplementary figure 3. Normal muscle function in 10-months old** ***Stim1^+/-^* males.** (A-D) At 10 months, WT and *Stim1^+/-^* mice manifested comparable maximal muscle force, muscle contraction and relaxation kinetics, and fatigue. (E) Tubular aggregates are scarce in WT tibialis anterior sections stained with Gomori trichrome (arrow) and absent in *Stim1^+/-^* mice.

**Supplementary figure 4. Abnormal mitochondrial biomarkers in** ***Stim1^+/-^* mice at 18 months.** (A-B) Western blots illustrating abnormal protein levels of diverse mitochondrial biomarkers in *Stim1^+/-^* tibialis anterior at 18 months. Protein levels of the cytosolic superoxide dismutase SOD1 were comparable in WT and *Stim1^+/-^* mice. Ponceau served as loading control. (C) Western blot and quantification of OXPHOS proteins revealed increased protein levels of mitochondrial complexes I, II, and V in *Stim1^+/-^* muscle compared with controls. Data are presented as mean values +/- SEM. T-test with Welch’s correction. Significant differences are indicated as **p<0.01 and ****p<0.0001.

| Gene | Forward | Reverse |
| --- | --- | --- |
| *Stim1* (genotyping) | AACGTCTTGCAGTTGCTGTAGGC | GGCTCTGCTGACCTGGAACTATAGTG |
| *Nd1* | AAGTTGATCGTAACGGAAGC | CCCATTCGCGTTATTCTT |
| *Cre* | GAACCTGATGGACATGTTCAGG | AGTGCGTTCGAACGCTAGAGCCTGT |
| *Rsp11* | CGCGTGGTGAATAAGGAAGC | GTAAGCACGCTCCGTCTGAA |
| *Pgc1α* | GCAGGTCGAACGAAACTGAC | CTTGCTCTTGGTGGAAGCAG |
| *Pan Stim1* | TGATTGAGGGGGTCCATCCA | AGCCAAGTGGGGAATTCGTG |
| *Stim1S* | TCAGTGCCGCTGTCACTTAG | CCCTCAGTGCCGCTGTCACT |
| *Stim1L* | CCCAACCCCTTCTGACAACA | ATGAAAGGCTACGCATTCGC |
| *Myh7* | CTACAGGCCTGGGCTTACCT | TCTCCTTCTCAGACTTCCGC |
| *Myh2* | ATCCAAGTTCCGCAAGATCC | TTCGGTCATTCCACAGCATC |
| *Myh1* | ATGAACAGAAGCGCAACGTG | AGGCCTTGACCTTTGATTGC |
| *Myh4* | AGACAGAGAGGAGCAGGAGAGTG | CTGGTGTTCTGGGTGTGGAG |

**Supplementary Table 1. List and sequences of genotyping and RT-qPCR primers**
